# Dynamic associations between stress and relationship functioning in the wake of COVID-19: Longitudinal data from the German family panel (pairfam)

**DOI:** 10.1177/02654075221092360

**Published:** 2022-11

**Authors:** Theresa Pauly, Janina Lüscher, Corina Berli, Urte Scholz

**Affiliations:** 1 Department of Psychology, 27217 University of Zurich, Zurich, Switzerland; 2 University Research Priority Program “Dynamic of Healthy Aging”, 27217 University of Zurich, Zurich, Switzerland

**Keywords:** Couples, relationship functioning, relationship quality, stress, COVID-19, german family panel, longitudinal analysis

## Abstract

Individuals all across the world experienced significant disruptions in their personal and family life with the outbreak of the new coronavirus disease 2019 (COVID-19). The current study investigated dynamic associations between stress and relationship functioning over time in the face of the COVID-19 pandemic. Perceived stress, relationship satisfaction, and relationship quality (appreciation, intimacy, conflict) were reported by 1483 young to middle-aged participants who were in a romantic relationship and lived with their partner in 2018/2019 and in May–July 2020 (a few months after the onset of COVID-19). Data were analyzed using bivariate latent change score models. Relationship functioning (satisfaction, appreciation, intimacy) showed small decreases from before to during the pandemic. Contrary to expectations, levels of perceived stress also decreased on average from before to during the pandemic. Changes in relationship functioning were correlated with changes in stress over time, so that participants with greater decreases in relationship satisfaction, appreciation, and intimacy and greater increases in conflict from before to during the pandemic showed lesser decreases/greater increases in stress. Higher pre-pandemic relationship satisfaction was associated with greater decreases/lesser increases in stress from before to during the pandemic. Pre-pandemic levels of other measures of relationship functioning or stress were not associated with changes in outcomes over time. Results add to the literature demonstrating that stress is closely intertwined with the functioning of intimate relationships. Furthermore, they suggest that greater relationship satisfaction may serve as a protective factor for stressful life events.

## Introduction

The new coronavirus disease 2019 (COVID-19) has resulted in soaring levels of stress and has put families under high pressure (Huebener et al., 2021; Prime et al., 2020). In particular, stay at home orders increased interactions with others in the same household such as romantic partners, which could have heightened partner influences on well-being in cohabiting couples (Pietromonaco & Overall, 2021). Romantic partners are closely linked in their mental and physical health (Kiecolt-Glaser & Wilson, 2017). In fact, links between marital quality and health outcomes are comparable in size to those with important lifestyle factors including exercise and diet (Robles et al., 2014). The partnership can act as a resource, supporting individuals to better manage stressful events (Kiecolt-Glaser & Wilson, 2017). Yet, a close interdependence with one’s partner also puts individuals at risk for stress transmission (Kiecolt-Glaser et al., 2020; Larson & Almeida, 1999). Previous research on life events such as terrorist attacks and natural disasters has shown that they have major potential to disrupt couple dynamics, for the good and bad (Cohan et al., 2009; Fredman et al., 2010; Marshall & Kuijer, 2017). In this paper, we examined how stress and relationship functioning (as indicated by overall relationship satisfaction and relationship quality) interacted over time in the face of the COVID-19 pandemic, drawing on data collected from young to middle-aged adults who lived with their partner in 2018/2019 and in the early stages of the pandemic (May–July 2020).

### Changes in stress and relationship functioning with the onset of COVID-19

According to the transactional model of stress (Lazarus & Folkman, 1984), perceived stress occurs when a person appraises that any internal or external demands placed on them exceed their capabilities to cope. COVID-19 has been accompanied with a number of challenges. This includes, but is not limited to, concerns about health and fear of dying, feelings of loneliness and lack of social contact, and financial strain (Tull et al., 2020; Wright et al., 2021). Thus, it is not surprising that studies have shown an increase in perceived stress and stress-related disorders with the onset of COVID-19 (McGinty et al., 2020; Pieh et al., 2021).

The pandemic has also resulted in significant changes to family life, such as one or both partners working from home, closures of daycares and schools necessitating childcare at home, and spending more time (when living together) or less time (when living apart) with the partner and less time with friends or other family members (Alzueta et al., 2021; Eales et al., 2021; Prime et al., 2020). COVID-19 related stress has been associated with lower relationship satisfaction and more conflict (Balzarini et al., 2020). Accordingly, there is preliminary evidence that relationship functioning has, on average, suffered with the onset of the pandemic (Goodboy et al., 2021; Schmid et al., 2021). However, we do not know how stress and relationship functioning were dynamically related over time, that is, how pre-pandemic stress was linked with subsequent changes to relationship functioning during COVID-19, and vice versa. In the following, we provide theoretical reasoning for both causal pathways.

### Dynamic associations between stress and relationship functioning over time

According to the Vulnerability-Stress-Adaptation model (Karney & Bradbury, 1995) pre-existing vulnerabilities interact with external stressors in predicting relevant interpersonal dynamics in couples (e.g., support, conflict management), which in turn influence the nature of changes in relationship quality over time, and ultimately determine relationship stability. Applying this model to the context of COVID-19, Pietromonaco and Overall (2021) suggest that the negative ramifications of COVID-related stressors on relationship functioning would be aggravated by vulnerabilities (located in the context or the individual) which existed before the pandemic. Specifically, they propose that couples whose coping resources were strained prior to the pandemic may find it more difficult to adaptively respond to any added stress due to COVID-19. Support comes from longitudinal studies demonstrating that individuals with higher stress report worse relationship quality over time and are more likely to divorce (Bodenmann, 1997; Bodenmann & Cina, 2006; Neff & Karney, 2004). Additionally, daily diary research shows that on days when individuals cope with heightened demands, social interactions are characterized by more conflict, withdrawal, and anger (Sears et al., 2016; Timmons et al., 2017). To sum up, individuals with higher pre-existing stress levels may lack the energy and resources needed to adaptively address later relationship difficulties (Neff & Karney, 2017), resulting in less healthy relationship functioning during COVID-19.

Empirical and theoretical literature also provides a rationale for the *opposite causal direction*, that is, that higher pre-pandemic relationship quality might act as a protective factor, being associated with lower stress levels during the pandemic. It has been shown that couples with high relationship satisfaction tend to combine their resources to tackle problems jointly: They more likely think of stressors, even those that only affect one individual, as “our” problem (Falconier & Kuhn, 2019; Lyons et al., 1998) and utilize positive dyadic coping strategies more often such as providing support, assuming responsibilities for the partner’s tasks, and engaging in joint and complementary efforts to deal with a stressor (Systemic-Transactional Model; Bodenmann, 1995). Accordingly, engaging in positive forms of dyadic coping has been linked with more effective individual coping strategies, increased well-being, and decreased psychological distress when coping with heightened stress, and when confronted with mental or physical health challenges (Falconier & Kuhn, 2019). To summarize, individuals who were in high quality relationships prior to COVID-19 might have been better equipped to effectively deal with pandemic-associated stress because they could rely on greater (dyadic) coping resources. Conversely, high marital distress prior to the pandemic might be indicative of dysfunctional relationship processes that could have put individuals at greater risk for later increased stress during the pandemic. As emphasized by the Dyadic Biobehavioral Stress Model (Shrout, 2021), negative relationship dynamics such as conflict and hostility can result in greater physiological stress reactivity, lower quality sleep (which can exacerbate stress), and heightened distress overall.

### The current study

Using data collected prior to and during the COVID-19 pandemic of 1483 individuals who lived with a romantic partner, the goal of this study was to test bidirectional relationships between stress and relationship functioning in the face of a major external stressor, the COVID-19 pandemic. We hypothesized that (H1) individuals report higher stress and worse relationship functioning (relationship satisfaction, relationship quality) during COVID-19, as compared to pre-pandemic levels. Furthermore, we assumed that (H2) pre-existing stress might act as a risk factor for worse later relationship functioning and that better pre-pandemic relationship functioning might act as a resource, protecting from increased stress during COVID-19. Hypotheses were pre-registered at https://osf.io/bjnzt.

## Methods

### Participants and procedure

**Figure 1.**
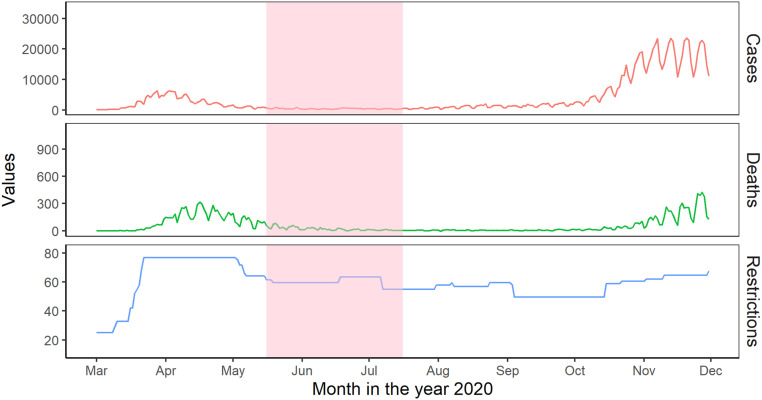
COVID-19 related cases, deaths, and governmental stringency in Germany in 2020. *Note.* The graph shows the development of COVID-19 related cases and deaths, as recorded by the Robert Koch-Institut, and governmental responses in Germany, as indicated by a stringency index developed by Hale et al. (2021). This index aggregates information on 20 pandemic policy indicators to indicate the number and stringency of restrictions (e.g., school closures, restrictions on gatherings, face coverings, and stay at home measures). The study period (T2) is highlighted in red.

Analyses are based on data from the 11th wave and the COVID-19 survey from the German Family Panel (pairfam; Brüderl et al., 2020; for a detailed description of study procedures and measures see Huinink et al., 2011; Walper et al., 2020). In the 11th pairfam wave, data were collected during an in-person, computer assisted interview, whereas data were collected using an online questionnaire in the COVID-19 pairfam survey. See Figure 1 for COVID-19 related cases, deaths, and governmental restrictions in the study period. Out of 1549 adults who completed the COVID-19 survey in May–July 2020 and lived with their romantic partner, 1526 individuals also provided data at the 11th wave which was collected in 2018 and 2019 (*M* time between surveys = 15.74 months, *SD* = 2.47). In this article, we focus on cohabiting couples because the pandemic might have posed unique and qualitatively distinct challenges to partners who do or do not live together (Vigl et al., 2022). In particular, partner influences might have been heightened in a situation when lockdown measures confined partners to the same living quarters, increasing time spent together. Information on stress or relationship functioning was missing for *n* = 43 individuals, resulting in a final sample of 1483 participants aged 24–48 years (*M* age = 36.9, *SD* = 7.2; 60% female). Participants mostly reported to be German natives without a migration background (85%), 5% had immigrated from a different country, 5% reported to be half-German, and 5% reported another non-German background. The majority of the sample identified as heterosexual (98%), 13 individuals (1%) identified as gay and 13 individuals (1%) identified as lesbian. Participants had 14.9 years of education, on average, (*SD* = 2.9, range: 8.0–20.0), had mostly been in a long-term relationship with their current partner (*M* = 12.2 years, *SD* = 7.8, range: 0–41.7), and lived with their partner for an average of 10.3 years (*SD* = 7.1, range: 0–30.1). The average net household income was 4169€ per month (*SD* = 2553). Ethics-approval for pairfam was granted by the ethics committee of the Faculty of Management, Economics, and Social Sciences of the University of Cologne. Informed consent was obtained from all participants included in the study. Power was >99% to detect small lagged associations (i.e., a standardized regression coefficient of .10; Cohen, 1992) between relationship functioning and stress over time.

### Measures

All item examples are translated from German.

*Relationship satisfaction*. Participants reported how satisfied they were with their relationship overall on a scale from 0 “very dissatisfied” to 10 “very satisfied” in the 11th pairfam wave (T1) and the COVID-19 pairfam survey (T2).

*Relationship quality*. Relationship quality was assessed using six items adapted from the Network of Relationships Inventory (Furman & Buhrmester, 1985) at T1 and T2. Specifically, participants were asked to rate how often certain things happen in their partnership on a scale of 1 “never” to 5 “always.” Two items each measured *appreciation* (“How often does your partner show that he/she appreciates you?”/“How often does your partner express recognition for what you've done?”), *intimacy* (“How often do you tell your partner what you're thinking?”/“How often do you share your secrets and private feelings with your partner?”), and *conflict* (“How often are you and your partner annoyed at or angry with each other?”/“How often do you and your partner disagree and quarrel?”). Responses were averaged to create a mean score for relationship quality (T1: α = .75; T2: α = .87). We further calculated scores for each of the three subscales, that is, appreciation (T1: *r* between the two items = .65; T2: *r* = .71), intimacy (T1: *r* = .57; T2: *r* = .65), and conflict (T1: *r* = .65; T2: *r* = .67).

*Stress*. Perceived stress was assessed at T1 and T2 using three items adapted from the Perceived Stress Questionnaire (Levenstein et al., 1993; German version by Fliege et al., 2009). In particular, participants reported to what extent they felt stressed, overburdened, or under pressure in the last four weeks on a scale of 0 “not at all” to 5 “absolutely” (T1: α = .87; T2: α = .86).

*Covariates.* Older age, male gender, shorter relationship duration, and having less young children have been linked with reports of better relationship functioning (Sorokowski et al., 2017; Wendorf et al., 2011). Furthermore, higher socio-economic status is tied to positive relationship and individual well-being outcomes (Conger et al., 2010). Finally, perceived stress during the pandemic was partly shaped by working conditions (such as home office, working full- or part-time) and perceived financial risk (Daly & Robinson, 2021; Rieth & Hagemann, 2021). Thus, we considered the following variables as covariates: age, gender, relationship duration, years of education, and number of persons aged <14 years in the household measured at T1 and a decrease of income due to COVID-19, both partners being employed full time, and both partners working from home measured at T2.

### Statistical analysis

We first tested our study outcomes for measurement invariance over time, in order to justify mean comparisons (Meredith, 1993). Specifically, we estimated models that constrained *loadings* of items to be equal for the first and second measurement point (weak measurement invariance). Then, we estimated models that constrained *loadings and intercepts* of items to be equal for the first and second measurement point (strong measurement invariance). Model fit was evaluated using the comparative fit index (CFI), the Tucker-Lewis index (TLI), the root mean square error of approximation (RMSEA), and the standardized root mean squared residual (SRMR). The following thresholds have been proposed to indicate good model fit: CFI >.95, TLI >.95, RMSEA <.06, SRMR <.08 (Hu & Bentler, 1999). Model fit indices for models testing measurement invariance can be found in Table 1. Our measure of stress showed strong measurement invariance over time, whereas the measurement models for relationship quality pointed to bad fit. However, model fit indices indicated strong measurement invariance for the three relationship quality subscales appreciation, intimacy, and conflict. Thus, in all subsequent models we investigated change in the three relationship quality facets over time, rather than using the aggregated relationship quality score.

**Table 1.** Longitudinal measurement invariance test for study outcomes.

	Stress		Relationship quality		Appreciation		Intimacy		Conflict	
Model fit index	Weak MI	Strong MI	Weak MI	Strong MI	Weak MI	Strong MI	Weak MI	Strong MI	Weak MI	Strong MI
CFI	.993	.993	.600	.596	.990	.990	.983	.984	.999	.999
TLI	.987	.992	.528	.577	>.999	.979	<.999	.969	>.999	.999
RMSEA	.052	.041	.196	.186	<.001	.077	<.001	.086	<.001	.020
SRMR	.016	.016	.133	.135	.012	.015	.030	.030	.005	.008

*Note*. *N* = 1483. MI = measurement invariance. CFI: comparative fit index; TLI: Tucker-Lewis index; RMSEA: root mean square error of approximation; SRMR: standardized root mean squared residual.

Then, we conducted bivariate latent change score models in Mplus Version 8.2 (Klopack & Wickrama, 2020; Muthén & Muthén, 1998-2017) to examine the bidirectional associations between changes in stress and changes in relationship functioning over time (Model A: relationship satisfaction, Model B: appreciation, Model C: intimacy, Model D: conflict). These models estimate individual values of within-person change in study outcomes as a latent variable (McArdle, 2009). In order to be able to interpret the intercept of the latent change variable as the estimated average change, we centered T1 and T2 stress and relationship functioning indices on their respective T1 mean (Coman et al., 2013). The autoregressive parameter from the T1 (pre-pandemic) measurement to the latent change factor denotes the extent to which the level of a given variable at T1 is associated with the magnitude of change that occurs in that variable between T1 and T2. Importantly, models allow to investigate a time-lagged coupling of the variables, that is, the extent to which change in one variable from before to during the pandemic (T1 to T2) is related to the pre-pandemic (T1) level in the other. Models also estimate the covariance of pre-pandemic levels and correlated change of the variables (after taking the coupling pathways into account). Furthermore, we tested whether time-lagged paths significantly differed from each other by comparing model fit (log-likelihood) of a model that constrained the time-lagged paths to be equal with an unrestricted model. Missings were treated as at random and models were estimated using full information maximum likelihood. Reported parameter estimates are standardized coefficients from models with standardized predictors and outcomes (STDYX), except for our exploratory follow-up analyses with respect to age group differences (binary predictor, only outcomes standardized; STDY). The model code is available online on the project’s OSF page (https://osf.io/pq95m/). Models control for covariates that showed at least small bivariate correlations (*r* = .10) with outcomes of interest at T1 or T2: age, relationship duration, and number of persons aged <14 years in the household^1^.

## Results

Table 2 shows sample descriptives and intercorrelations between central study variables and included covariates (please see S-Table 1 in the Supplemental material for a correlation table with all measured covariates). Measures of relationship functioning showed large correlations of pre-pandemic scores with scores during the pandemic (*r* = .48 to .61). Pre-pandemic stress was moderately correlated with pandemic stress levels (*r* = .28).

**Table 2.** Descriptives and intercorrelations of central study variables (*N* = 1483 participants).

Variable	*M* (SD)	2	3	4	5	6	7	8	9	10	11	12	13
1. Relationship satisfaction T1	8.04 (1.92)	.48**	.38**	.29**	.33**	.26**	-.39**	-.29**	-.11**	-.10**	-.12**	-.07*	-.10**
2. Relationship satisfaction T2	7.74 (1.78)		.41**	.56**	.33**	.51**	-.32**	-.45**	-.08**	-.18**	-.12**	-.07**	-.07**
3. Appreciation T1	3.82 (0.73)			.61**	.42**	.38**	-.35**	-.29**	-.06*	-.07**	-.16**	-.22**	-.21**
4. Appreciation T2	3.60 (0.76)				.30**	.51**	-.29**	-.36**	-.07**	-.13**	-.10**	-.12**	-.15**
5. Intimacy T1	3.78 (0.72)					.59**	-.20**	-.11**	.03	-.00	-.24**	-.19**	-.10**
6. Intimacy T2	3.67 (0.79)						-.18**	-.21**	.00	-.06*	-.18**	-.14**	-.12**
7. Conflict T1	2.49 (0.62)							.60**	.12**	.08**	-.01	.05*	.10**
8. Conflict T2	2.51 (0.65)								.11**	.21**	-.02	.00	.08**
9. Stress T1	3.06 (1.04)									.28**	-.04	-.02	.03
10. Stress T2	2.81 (1.12)										-.00	.02	.22**
11. Age	36.88 (7.18)											.64**	.21**
12. Relationship duration (years)	12.22 (7.79)												.27**
13. Number of persons aged < 14 years in household	0.94 (1.01)												

*Note*. Relationship satisfaction scored from 0 “very dissatisfied” to 10 “very satisfied.” Items for the three relationship quality facets (appreciation, intimacy, conflict) were rated on a scale from 1 to 5. Perceived stress was measured on a scale of 0 “not at all” to 5 “absolutely.” Measures at T1 (pre-pandemic) were collected in 2018/2019; measures at T2 (during the pandemic) were collected from May to July 2020.

*p < .05. ** *p* < .01.

### Changes in stress and relationship functioning with the onset of COVID-19

**Figure 2.**
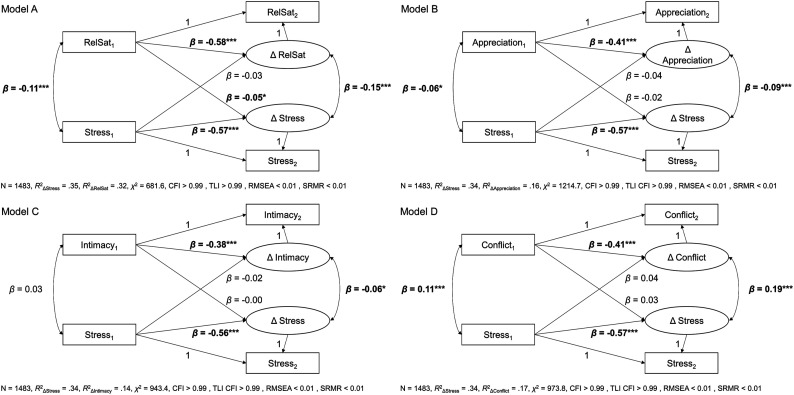
Bivariate latent change score models for the associations of relationship satisfaction and relationship quality facets with stress over time. *Note*. The figure shows results from bivariate latent change score analyses of relationship satisfaction/three relationship quality facets and stress over time, pre COVID-19 to during COVID-19. RelSat = Relationship satisfaction. The subscripts 1 and 2 indicate time 1 (pre-pandemic) and time 2 (during pandemic). Covariates (age, relationship duration, and number of persons aged <14 years in the household) are not depicted in the figure for simplification. *β*s are standardized estimates. Bold font denotes significant coefficients. * *p* < .05. ** *p* < .01. *** *p* < .001.

Bivariate latent change score models for hypothesized bidirectional associations of stress with relationship satisfaction and the three relationship quality facets are depicted in Figure 2. Full model results including covariates can be found on the project’s OSF page (https://osf.io/pq95m/). Contrary to our hypothesis (H1), participants reported lower stress during the pandemic (at T2), as compared to before the pandemic (at T1; *β* = −0.19, SE = 0.02, *p* < .001). As expected (H1), participants reported lower relationship satisfaction during the pandemic, as compared with before the pandemic (*β* = −0.16, SE = 0.02, *p* < .001). With respect to the three relationship quality facets, there was no significant change in conflict from before to during the pandemic (*β* = 0.02, SE = 0.02, *p* = .463), whereas appreciation (*β* = −0.33, SE = 0.03, *p* < .001) and intimacy (*β* = −0.17, SE = 0.02, *p* < .001) decreased from before to during the pandemic.^2^ Participants showed significant unexplained differences in the extent to which stress (*σ*^2^ = 0.66, *p* < .001), relationship satisfaction (*σ*^2^ = 0.68, *p* < .001), appreciation (*σ*^2^ = 0.84, *p* < .001), intimacy (*σ*^2^ = 0.86, *p* < .001), and conflict (*σ*^2^ = 0.84, *p* < .001) changed from T1 to T2.

### Dynamic associations between stress and relationship functioning over time

**Figure 3.**
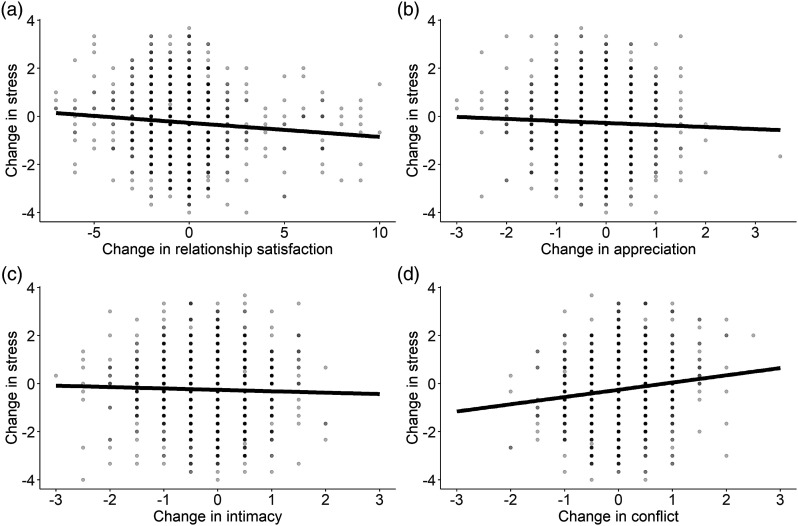
Correlated change in relationship functioning and stress from pre-COVID-19 levels to levels during COVID-19. *Note*. The figures show bivariate Pearson correlations between changes in stress and relationship functioning from before the pandemic (2018/2019) to during the pandemic (May–July 2020). Greater decreases in relationship satisfaction (a), appreciation (b), and intimacy (c), and greater increases in conflict (d) over time were associated with a greater increase/lesser decrease in stress from before to during COVID-19.

Model A examined hypothesized associations between relationship satisfaction and stress over time (H2). Stress before the pandemic (at T1) significantly co-varied with relationship satisfaction before the pandemic (*β* = −0.11, *SE* = 0.03, *p* < .001). Accounting for time-lagged associations, changes in stress (from T1 to T2) were significantly associated with changes in relationship satisfaction (*β* = −0.15, *SE* = 0.03, *p* < .001, Figure 3). The time-lagged paths were significant in one direction for Model A: Higher pre-pandemic relationship satisfaction predicted greater decreases/lesser increases in stress during the pandemic (*β* = −0.05, *SE* = 0.02, *p* = .033). Higher pre-pandemic stress was not related to changes in relationship satisfaction from before to during the pandemic (*β* = −0.03, SE = 0.02, *p* = .136). However, a model constraining the lagged paths to be equal did not fit worse than a model freely estimating the lagged parameters (*χ*^2^ (1) = 0.43, *p* = .512). Model A explained 32% of variance in change in relationship satisfaction (*p* < .001) and 35% in change in stress (*p* < .001).

As a next step, we tested links between the three relationship quality facets and stress over time (H2, Models B to D for appreciation, intimacy, conflict; see Figure 3). Higher pre-pandemic stress was not significantly associated with pre-pandemic intimacy (*β* = 0.03, *SE* = 0.03, *p* = .286), but was associated with lower pre-pandemic appreciation (*β* = −0.06, *SE* = 0.03, *p* = .018) and higher pre-pandemic conflict (*β* = 0.11, *SE* = 0.03, *p* < .001). Accounting for time-lagged paths, changes from before to during the pandemic in all three relationship quality facets were associated with concurrent changes in stress from T1 to T2 (see Figure 3; Δ stress and Δ appreciation: *β* = −0.09, *SE* = 0.03, *p* < .001; Δ stress and Δ intimacy: *β* = −0.06, *SE* = 0.03, *p* = .032; Δ stress and Δ conflict: *β* = 0.19, *SE* = 0.03, *p* < .001). Findings with respect to time-lagged parameters did not provide evidence for a bidirectional association between the relationship quality facets and stress over time. Thus, pre-pandemic relationship quality did not predict later change in stress or vice versa. Models explained 16% of variance in change in appreciation, 14% of variance in change in intimacy, 17% of variance in change in conflict, and 34% of variance in change in stress (all *p* < .001).

### Exploratory follow-up analyses on age differences

In follow-up analyses, we explored whether the found associations differed by age cohort (*n* = 509 younger adults aged 24–35 years; *n* = 974 middle-aged adults aged 36–48 years). We found that relationship satisfaction (*β* = −0.09, *SE* = 0.05, *p* = .039) and intimacy (*β* = −0.13, *SE* = 0.05, *p* = .011) showed a more pronounced decrease from before to during the pandemic in middle-aged, as compared with younger adults. Changes in appreciation and conflict did not differ by age group and we found no evidence for age differences in time-lagged associations.

## Discussion

In the year of 2020, individuals were confronted with significant challenges to their social and mental well-being (Alzueta et al., 2021). These circumstances presented a unique opportunity to investigate the importance of pre-pandemic stress and relationship functioning for later changes in stress and relationship functioning during COVID-19. In doing so, we tested predictions based on two relationship theories, which emphasize pre-existing stress as a risk factor for worse relationship outcomes (Karney & Bradbury, 1995) and pre-existing positive relationship quality as a resource to better cope with external demands (Bodenmann, 1995). As compared with pre-pandemic levels, our sample of individuals cohabiting with their partner reported lower stress, lower relationship satisfaction, lower appreciation, and lower intimacy during the pandemic. We also found that individuals who reported higher pre-pandemic relationship satisfaction showed greater decreases/lesser increases in stress from before to during COVID-19. No other pre-pandemic relationship functioning measure was associated with later change in stress, nor was higher pre-pandemic stress linked with later change in relationship functioning. However, we found evidence for correlated change in stress with all four relationship functioning measures (relationship satisfaction, appreciation, intimacy, conflict) from before to during the pandemic.

### Changes in stress and relationship functioning with the onset of COVID-19

In contrast to other studies (McGinty et al., 2020), we found that perceived stress was lower during COVID-19 in our sample, as compared to pre-pandemic levels. The difference, however, was relatively small. This might be explained by the timing of the study (May-July 2020). Research shows that COVID-related distress seemed to wane over time, as reflected in initially high stress levels in March/April 2020 and a subsequent decline (Zacher & Rudolph, 2021). This drop could partly be explained by decreases in perceived health risk, perceived financial risk, and lifestyle restrictions (Bönisch et al., 2020; Robinson & Daly, 2021). Thus, we may not have captured individuals’ peak stress response to the pandemic. Furthermore, all of our participants were in a romantic relationship and prior research has shown that individuals who were single were at higher risk for distress during the pandemic, as compared with married individuals (Kowal et al., 2020). Another possible explanation may be that the pandemic changed subjective stress appraisals by putting stressful experiences into perspective (Fernández Cruz et al., 2020). A burgeoning literature points to the ability of humans to grow in the face of disasters and other major stressful life events (Shing et al., 2016; Wu et al., 2019). Specifically, being faced with an uncontrollable and potentially life-threatening disease, the appraised harm of daily hassles and consequently their impact on one’s well-being might fade (Shing et al., 2016).

Similarly, changes in relationship functioning were relatively small in our sample of cohabiting partners, and significantly differed between participants. Other researchers have predicted and shown a large variability in COVID-19 related impact on relationships and family life, with some relationships experiencing turbulence and others growing stronger (Eales et al., 2021; Prime et al., 2020; Williamson, 2020). A salience of the fragility of life might prompt individuals to turn to close others to seek connection, security, and comfort (Marshall & Kuijer, 2017; Mikulincer & Shaver, 2007). In line with this idea, studies have associated other life events such as terrorist attacks and natural disasters with increased relationship quality and reduced divorce rates (Cohan et al., 2009; Fredman et al., 2010; Nakonezny et al., 2004). We found that, on average, individuals reported lower relationship satisfaction, lower appreciation, and lower intimacy during COVID-19, as compared with pre-COVID levels. This is in line with a large cross-national study (data from 68 countries) reporting a general decline in relationship satisfaction using retrospective ratings for pre-pandemic levels (Vigl et al., 2022). In contrast, relationship conflict did not significantly change over time. One reason might be that levels of conflict were generally relatively low in the current sample and that the measured construct was less sensitive to capture change. Furthermore, it might be that the pandemic specifically impacted relationship functioning in that it decreased positive relationship qualities but that it did not necessarily increase negative relationship qualities (Ahuja & Khurana, 2021; Ross et al., 2019). It has been hypothesized that partners engaged in more conflict avoidance during the pandemic because they feared relationship dissolution in times of uncertainty and that the confrontation with a number of other threats made individuals place lesser weight on relationship problems (Li & Samp, 2021).

In follow-up analyses, we found that decreases in relationship satisfaction and intimacy were stronger in middle-aged as compared with younger adults. Couples in midlife, especially those that were caring for young children during the pandemic full time, might have experienced particular difficulties to make space or time for shared activities that build intimacy and closeness (Pietromonaco & Overall, 2021). Additionally, middle-aged individuals often balance multiple roles and goals (e.g., pursuing a career, caring for children and aging parents) while at the same time being confronted with the onset of age-related declines in cognition and health (Infurna et al., 2020). These factors may make midlife a time in life when individuals are more vulnerable to negative ramifications of stressful life events (i.e., the pandemic) on relationship functioning.

### Dynamic associations between stress and relationship functioning over time

We found some evidence for the assumption that pre-pandemic positive relationship functioning might act as a resource for warding off later stress (Systemic-Transactional Model, Bodenmann, 1995). Higher pre-pandemic relationship satisfaction (but not higher pre-pandemic appreciation, higher pre-pandemic intimacy, or lower pre-pandemic conflict) was associated with greater decreases/lesser increases in stress during the pandemic. Higher pre-pandemic satisfaction might be linked with an individual’s positive appraisal of coping resources (e.g., support from their partner) to tackle pandemic-related challenges, resulting in lower perceived stress during COVID-19. Results dovetail with findings by Donato et al. (2020), who showed that higher relationship satisfaction was linked with more stress communication and more dyadic coping during the pandemic, and that greater dyadic coping responses were associated with better psychological well-being. A reason for the non-significant finding between pre-pandemic relationship quality indicators and later change in stress could be that specific facets of the relationship to the partner such as appreciation, intimacy, and conflict might be more closely related to affective well-being (Kansky, 2018), rather than the appraisal of coping resources. Thus, future research could build on the current findings by examining the relative importance of relationship quality facets for later subjective well-being during COVID-19, e.g., for decreased positive affect, increased negative affect, and decreased life satisfaction (Anglim & Horwood, 2021).

Pre-pandemic stress levels were not associated with changes in relationship satisfaction, appreciation, intimacy, or conflict from before to during the pandemic. Thus, we did not find support for the notion that pre-pandemic stress acted as a risk factor for the erosion of positive relationship functioning in the wake of COVID-19. This dovetails with prior research observing stronger effects from relationships on mental health and individual well-being, than vice versa (Braithwaite & Holt-Lunstad, 2017; Proulx et al., 2007). However, the pandemic strained some resources more than others (e.g., financial resources, interpersonal resources; Tull et al., 2020; Wright et al., 2021). For example, Balzarini et al. (2020) reported that greater loneliness and financial strain at the onset of COVID-19 were associated with lower relationship satisfaction and greater relationship conflict. Thus, it is also conceivable that findings might differ by the type of pre-pandemic stress, with pre-existing demands in some areas (e.g., mental health challenges or interpersonal demands such as being a caregiver for aging parents) being more strongly related to later relationship functioning than others (e.g., job demands).

In sum, we did not find strong evidence for the hypothesized bidirectional associations of pre-pandemic relationship functioning and stress with later change in relationship functioning and stress during the pandemic. Instead of initial levels acting as vulnerabilities or protective factors, findings rather speak to a dynamic linkage of changes in stress with relationship satisfaction and relationship quality indicators over time. Accordingly, individuals who experienced greater decreases in relationship satisfaction, appreciation, and intimacy and greater increases in conflict over time reported a greater increase/lesser decrease in stress from before to during the pandemic.

### Strengths, limitations, and future directions

This study linked data on stress and relationship functioning collected in the midst of the pandemic to pre-pandemic levels, in a large sample of young and middle-aged adults (midlife tends to be understudied in psychological research; Infurna et al., 2020). As a limitation our sample overall reported relatively high relationship functioning. Thus, we do not know if findings generalize to couples with lower relationship quality. Furthermore, we focused on individuals who cohabit with their partner and our findings might not generalize to non-cohabiting couples. Findings from Vigl et al. (2022) emphasize that the pandemic might have differentially impacted partners depending on their living situation, showing that relationship satisfaction showed greater decreases for non-cohabiting, as compared with cohabiting partners, and that joint activities and physical intimacy increased and time for oneself decreased for cohabiting partners, whereas non-cohabiting individuals showed the reverse pattern. Utilizing bivariate latent change score models, we were able to examine coupled associations of study outcomes measured prior to the pandemic with later change in these outcomes. Yet, although the longitudinal design can provide some evidence for temporal precedence, causality cannot be established. The COVID-19 survey of the pairfam only collected data from anchor participants but not their partners. Thus, we were only able to consider one partner’s perspective in the current manuscript. A dyadic approach to stress and relationship functioning that takes both partners’ perspectives into account is an important extension of the present work (Shrout, 2021). The pairfam is a prospective study and will continue collecting data of the present sample as well as their romantic partners. Future studies could build on the current findings by examining what kind of couples experience a recovery of their relationship functioning after the pandemic and by identifying risk factors for relationship dissolution. Finally, we used a single-item measure for relationship satisfaction and a 6-item measure for relationship quality (two items per subfacet). Future research needs to examine the bidirectional associations between stress and relationship functioning using more comprehensive measures of relationship dynamics.

## Conclusion

The year of 2020 has brought about major challenges for intimate relationships. On average, perceived stress, relationship satisfaction, appreciation, and intimacy showed small decreases from before to during the pandemic in our sample of partnered cohabiting young to middle-aged adults. We also found that longitudinal decreases in relationship satisfaction and intimacy were particularly salient in middle-aged, as compared with younger adults. Furthermore, this study provided evidence for a close linkage of changes in stress and changes in relationship functioning over time. Less support was found for our hypothesis that initial levels of stress might act as a vulnerability and that initial levels of relationship functioning might act as a resource for change in stress and relationship functioning from before to during the pandemic. One exception was that higher pre-pandemic relationship satisfaction was related to lesser increases/higher decreases in stress during the pandemic.

## Supplemental Material

Supplemental material - Dynamic associations between stress and relationship functioning in the wake of COVID-19: Longitudinal data from the German family panel (pairfam) Click here for additional data file. Supplemental material for Dynamic associations between stress and relationship functioning in the wake of COVID-19: Longitudinal data from the German family panel (pairfam) by Theresa Pauly, Janina Lüscher, Corina Berli, and Urte Scholz in Journal of Social and Personal Relationships

## Notes

1. We further ran models controlling for cohabitation duration instead of relationship duration. All findings replicate.

2. Results compare to t-tests for dependent samples (stress: *M*_diff_ = −0.25, *t*(1482) = −7.40, *p* < .001, Hedges’s *g* = 0.2; relationship satisfaction: *M*_diff_ = −0.30, *t*(1482) = −6.11, *p* < .001, Hedges’s *g* = 0.16; appreciation: *M*_diff_ = −0.22, *t*(1478) = −12.62, *p* < .001, Hedges’s *g* = 0.30; intimacy: *M*_diff_ = −0.12, *t*(1480) = −6.65, *p* < .001, Hedges’s *g* = 0.15; conflict: *M*_diff_ = 0.01, *t*(1481) = 0.66, *p* = .508, Hedges’s *g* = 0.02).
