# Supplementary material for: Dynamic associations between stress and relationship functioning in the wake of COVID-19: Longitudinal data from the German family panel (pairfam)
Source: J Soc Pers Relat. 2022 Nov;39(11):3183–203. doi: 10.1177/02654075221092360 (PMC9047667; doi:10.1177/02654075221092360)
Supplement: Supplemental material - Dynamic associations between stress and relationship functioning in the wake of COVID-19: Longitudinal data from the German family panel (pairfam) [file sj-pdf-1-spr-10.1177_02654075221092360.pdf]

S-Table 1

Descriptives and Intercorrelations of Study Variables (N = 1483 participants)

Variable	*M* (*SD*) or %	2	3	4	5	6	7	8	9	10	11	12	13	14	15	16	17	18
**1. Relationship satisfaction T1**	8.04 (1.92)	.48**	.38**	.29**	.33**	.26**	-.39**	-.29**	-.11**	-.10**	-.12**	-.07*	-.10**	.00	0.01	0.03	.04	-.02
**2. Relationship satisfaction T2**	7.74 (1.78)		.41**	.56**	.33**	.51**	-.32**	-.45**	-.08**	-.18**	-.12**	-.07**	-.07**	.00	0.04	0.00	-.03	-.02
**3. Appreciation T1**	3.82 (0.72)			.61**	.42**	.38**	-.35**	-.29**	-.06*	-.07**	-.16**	-.22**	-.21**	-.03	0.00	0.03	.08**	-.03
**4. Appreciation T2**	3.60 (0.76)				.30**	.51**	-.29**	-.36**	-.07**	-.13**	-.10**	-.12**	-.15**	-.04	0.00	0.01	.04	-.03
**5. Intimacy T1**	3.78 (0.72)					.59**	-.20**	-.11**	.03	.00	-.24**	-.19**	-.10**	.17**	0.01	.07*	.01	.01
**6. Intimacy T2**	3.67 (0.79)						-.18**	-.21**	.00	-.06*	-.18**	-.14**	-.12**	.12**	0.01	0.04	-.01	-.02
**7. Conflict T1**	2.49 (0.62)							.60**	.12**	.08**	-.01	.05*	.10**	.02	0.02	0.02	-.05	.03
**8. Conflict T2**	2.51 (0.65)								.11**	.21**	-.02	.00	.08**	.06*	0.00	0.04	-.05	.04
**9. Stress T1**	3.06 (1.04)									.28**	-.04	-.02	.03	.01	-.02	.06*	.02	.06*
**10. Stress T2**	2.81 (1.12)										.00	.02	.22**	.08**	0.00	.05*	.00	.05*
**11. Age**	36.88 (7.18)											.64**	.21**	-.05*	-0.02	-0.01	-.06*	-.02
**12. Relationship duration**	12.22 (7.79)												.27**	.07*	0.00	-0.01	-.11**	-.02

**(years)**																		
**13. Number of persons aged < 14 years in household**	0.94 (1.01)													.02	0.00	.10**	-.23**	-0.01
**14. Gender (1 = female)**	60% female														0.01	0.01	-.08**	0.02
**15. COVID-related decrease in income**	30%															-.08**	-.17**	-0.02
**16. Years of education**	14.90 (2.96)																-0.02	.19**
**17. Partners employed full-time**	27%																	.06*
**18. Partners in home office**	8%																	

Note. Relationship satisfaction scored from 0 “very dissatisfied” to 10 “very satisfied”. Items for the three relationship quality facets (appreciation, intimacy, conflict) were rated on a scale from 1 to 5. Perceived stress was measured on a scale of 0 “not at all” to 5 “absolutely”. Measures at T1 (pre-pandemic) were collected in 2018/2019; measures at T2 (during the pandemic) were collected from May to July 2020.

* $p < .05$. ** $p < .01$.
